# Screening for inter-hospital differences in cesarean section rates in low-risk deliveries using administrative data: An initiative to improve the quality of care

**DOI:** 10.1186/1472-6963-8-3

**Published:** 2008-01-04

**Authors:** Willem Aelvoet, Francis Windey, Geert Molenberghs, Hans Verstraelen, Patrick Van Reempts, Jean-Michel Foidart

**Affiliations:** 1 Federal Service of Health, Food Chain Safety and Environment, Brussels, Belgium; 2 Epidemiologie en Sociale Geneeskunde, Universiteit Antwerpen, Antwerp, Belgium; 3 Faculteit Geneeskunde en Farmacie, Vrije Universiteit Brussels, Brussels, Belgium; 4 Center for Statistics, Hasselt University, Diepenbeek, Belgium; 5 Department of Obstetrics & Gynaecology, Ghent University, Ghent University Hospital, Ghent, Belgium; 6 Department of Neonatology, Antwerp University Hospital, Antwerp, Belgium; 7 Department of Obstetrics and Gynaecology, La Citadelle Hospital, University of Liège, Liège, Belgium

## Abstract

**Background:**

Rising national cesarean section rates (CSRs) and unexplained inter-hospital differences in CSRs, led national and international bodies to select CSR as a quality indicator. Using hospital discharge abstracts, we aimed to document in Belgium (1) inter-hospital differences in CSRs among low risk deliveries, (2) a national upward CSR trend, (3) lack of better neonatal outcomes in hospitals with high CSRs, and (4) possible under-use of CS.

**Methods:**

We defined a population of low risk deliveries (singleton, vertex, full-term, live born, <4500 g, >2499 g). Using multivariable logistic regression techniques, we provided degrees of evidence regarding the observed departure ([relative risk-1]*100) of each hospital (N = 107) from the national CSR and its trend. To determine a benchmark, we defined three CSR groups (high, average and low) and compared them regarding 1 minute Apgar scores and other neonatal endpoints. An anonymous feedback is provided to the hospitals, the College of Physicians (with voluntary disclosure of the outlying hospitals for quality improvement purposes) and to the policy makers.

**Results:**

Compared with available information, the completeness and accuracy of the data, regarding the variables selected to determine our study population, showed adequate. Important inter-hospital differences were found. Departures ranged from -65% up to +75%, and 9 "high CSR" and 13 "low CSR" outlying hospitals were identified. We observed a national increasing trend of 1.019 (95%CI [1.015; 1.022]) per semester, adjusted for age groups. In the "high CSR" group 1 minute Apgar scores < 4 were over-represented in the subgroup of vaginal deliveries, suggesting CSs not carried out for medical reasons. Under-use of CS was also observed. Given their questionable completeness, except Apgar scores, our neonatal results, showing a significant association of CS with adverse neonatal endpoints, are to be cautiously interpreted. Taking the available evidence into account, the "Average CSR" group seemed to be the best benchmark candidate.

**Conclusion:**

Rather than firm statements about quality of care, our results are to be considered a useful screening. The inter-hospital differences in CSR, the national CS upward trend, the indications of over-use and under-use, the geographically different obstetric patterns and the admission day-related concentration of deliveries, whether or not by CS, may trigger initiatives aiming at improving quality of care.

## Background

Over the past few decades, there has been a tremendous rise in the number of deliveries performed through cesarean section in most industrialized countries. While both longitudinal as well as cross-sectional variations in cesarean section rates would be expected to reflect primarily differences in obstetric complications, it is actually observed that wide differences occur between countries, regions or even hospitals within the same region with similar socio-economic profiles and patient characteristics [1,2]. The latter seems to suggest that CS is probably often performed for non-medical reasons leading to an overall overuse of this surgical obstetric intervention. Indeed, it has been acknowledged that elective primary and repeat CS contributed heavily to the cesarean rise [3,4]. In the US for instance, the overall CSR increased by some 14% from 1998 to 2001 as a result of a 13% increase in medically indicated primary CS, yet a 53% increase in the rate of elective primary CS [3]. Similarly, vaginal birth after cesarean delivery (VBAC) rates decreased by 27% between 1996 and 2000, because of the rare but potentially catastrophic risks and medical litigation [4,5].

Meikl et al[3] describe elective caesarean as:" Elective cesarean deliveries can include medically and obstetrically indicated procedures that generally occur before labor. Elective cesarean deliveries can also include procedures for which there is no clear medical or obstetric indication." In the framework of a quality indicator, which aims at to monitor and reduce the caesarean rate [1], both aspects of their description are to be considered.

When comparing the CSRs between hospitals it is then important to exclude from the comparison medically and obstetrically indicated cesarean sections[4] and only to include procedures for which there is no clear medical or obstetric indication. We will call the latter type of cesarean sections: "elective cesareans" in the remainder of this manuscript. We also consider a repeat cesarean as medically and obstetrically indicated and therefore to be excluded from the comparison.

It is difficult to strike a balance between elective CS, which we understand as a procedure that occurred before labor, and vaginal delivery in the absence of randomized controlled trials [2].

Yet, it appears to be well established that in case of uncomplicated pregnancy, cesarean section exposes the parturient to inadvertent risks without offering a defined benefit [6]. The increase in the incidence of placenta accreta (from 1/30,000 pregnancies in the 1950s to 1/533 nowadays) and its dramatic consequences mainly have been ascribed to the increased CSR [7]. As a matter of fact, several studies revealed that the overall increase in cesarean rates has not led to a general decrease in perinatal mortality or birth asphyxia [8,9] and some countries with rather low CSRs experience low perinatal mortality rates, suggesting that good perinatal outcome does not necessarily equate high CSRs [10]. Nonetheless some authors argue that elective CS is as safe as or even safer than vaginal delivery [11].

Financial reasons and relative easiness may play a part in the mode of delivery decision-making, and when analyzing data on the subject, it can be difficult to distinguish between patient's or physician's choice [2,6,12]. Informed consent, maternal preferences, maternal autonomy, and the physician factor may play a role as well. Finally, decreasing parity (most women have less than two pregnancies) and medico-legal considerations can also contribute to the decision process [6,11,13].

Though it proves difficult to pinpoint the adequacy of obstetric care at the patient level. There is a defined need to monitor cesarean rates at the national, regional, and hospital levels to detect both over- and under-use[10] of CS. The use of CSR as a quality indicator in some countries, the contribution of informed consent and societal considerations to the decision-making process as well as clinical and epidemiological considerations, and the knowledge that CRS can be safely reduced by targeted interventions, led us to use CSR as a quality indicator of health care [1,14,15]. As CSR is a process indicator rather than an outcome itself, we also tried to demonstrate that CSR affects neonatal outcomes [16].

In the present population-based study, we used hospital discharge abstracts to select a predefined population of low risk deliveries and we subsequently aimed to explore the presence of both statistically and clinically significant inter-hospital differences in hospital-specific CSRs. We further hypothesized that hospitals with a high CSR would not experience better neonatal outcomes. Possible under-use was also to be evaluated.

## Methods

### Data sources

Since October 1990 all Belgian hospitals are subjected to compulsory registration with the health authorities of each admission through a standard form containing a defined set of clinical data including ICD-coded diagnoses and procedures. These discharge abstracts are termed Minimal Clinical Data (MCD) and contain patient data (among which year of birth, gender, residence, and anonymous hospital and patient identifiers), stay data (among which year and month of admission and discharge, length of stay, transfer to another hospital with specification of the type of hospital) and an unlimited number of diagnoses and procedures. This information is transmitted to the authorities for compilation and processing. Hospitals are further characterized according to teaching status (teaching or non-teaching), ownership (private or public) and the presence or absence of intensive maternal or neonatal care or otherwise. Hence, the MCD database covers all stays in Belgian hospitals, including those of non-residents. Because of the absence of a unique patient identifier, it is not possible, in case of a neonatal transfer, to get matching maternal and infant data in the "intake" hospital. Conversely all these data are available in the "discharge" facility's data.

In order to assess possible incompleteness and/or inaccuracy of the data – which are well-known drawbacks of administrative data [17] – we compared the MCD data with various partially overlapping registries, in particular data extracted from (1) the National Institute of Statistics (NIS), which are confined to residents, (2) the Center for the Study of Perinatal Epidemiology (SPE), a population-based perinatal data registry, confined to Flanders, the Northern half of the country, and (3) published data from the Office de la Naissance et de l'Enfance (ONE), providing perinatal data regarding the Southern part of the country [18].

In the SPE registry all perinatal deaths and live births occurring in the participating Flemish obstetrical units (residents and non-residents) are recorded, as well as a proportion of the home deliveries (about 1% of all deliveries). The data are collected on a routine and continuous basis and are submitted to an organized system of quality insurance [19]. The NIS data may be considered almost complete and of good quality as these data originate from the register of births, deaths and marriages, frequently used for administrative purposes. The ONE data regarding Apgar score and birth weight may be considered complete [20].

### Definition of the study population

A valid inter-hospital comparison of CSRs requires an adjustment for case mix. Alternatively, a population with a presumably equal risk of obstetric intervention may be defined for further analysis. Accordingly, we aimed to define a subgroup of parturients which is considered at low-risk of having a medically indicated CS [21], by excluding any patient that could have been considered at increased risk for a CS according to the US Agency for Healthcare Research and Quality (AHRQ) [1].

We excluded all deliveries involving abnormal presentation (including breech) or breech procedure, preterm gestation (< 37 weeks), fetal death, and multiple gestations according to the criteria of the US AHRQ. On request of the Belgian College of Physicians we further excluded all cases of full-term small-for-gestational age (defined as newborns born after 36 completed weeks of gestation with a birth weight of less than 2500 g) or macrosomia (defined as by birth weight of at least 4500 g).

All above-mentioned maternal characteristics were identified by Diagnosis Related Group (DRG) or by diagnosis and procedure codes of the International Classification of Diseases 9^th ^Revision Clinical Modification (ICD-9-CM). (Precise ICD-9-CM diagnosis and procedure codes can be found on the AHRQ's website [3].

The sampling frame consisted of the 455,933 deliveries, involving live born singletons in vertex presentation that were registered from 2001 to 2004. Thereof 86,310 (18.96%) were classified as cesarean deliveries. By applying the aforementioned criteria the final data set, the *study population*, comprised 381,989 deliveries of which 49,578 (12.98%) by cesarean section. Out of the 73, 944 deliveries, not meeting these criteria, 36,732 (49,68%) were cesarean deliveries, the *comparison population*.

### Analysis

Our aim was to identify, on the one hand, hospitals, with higher quality of obstetric care for benchmark and exemplary function purposes, and, on the other hand, hospitals with lower quality of care in order to help them improve their processes.

To assess hospital-specific rates of CS relative to the overall CS rate two analyses were carried out: a cross-sectional one focusing on the CSRs for the entire time span of the study (we call it the period) and a longitudinal one (we call it the trend) focusing on the per-semester evolution of the CSRs. In the latter analysis the unit of time used is the semester, the first semester comprising the first six months of the calendar year.

It has been suggested that in analyses, founded on administrative databases, confounding cannot be ruled out as an explanation of rather small, yet statistically significant effect sizes, such as a relative risk (RR) of 0.75 [22]. Therefore we defined a zone of non-interpretation, where the CS rate or trend of a hospital, compared with the national ones, should not be described as being "higher" or "lower". To determine the boundaries of this zone we firstly computed per hospital the relative risk (RR) of a hospital of having a higher/lower CS rate or trend than the national the national ones. We then calculated a departure D (expressed in %): with the formula D = (RR-1) × 100. Subsequently we defined the lower boundary as corresponding to a departure of minus 25 – which is equivalent to the afore-mentioned RR of 0.75 – and the upper boundary as a departure of plus 35, the lower boundary's approximate, statistical counterpart.

In the absence of any references regarding the significance of departures from the CS trend and by assuming that data quality has remained constant over time, a similar zone of non-interpretation was defined to allow for a comparison in the evolution over time in the hospital-specific CS rates by which we arbitrarily allowed for a -5 to +5 departure from the national trend. In the other cases, characterized by important departures, the results of the analysis were interpreted according to the degree of statistical evidence. We labeled this evidence 1) "strong" if the probability of finding a departure, as important or bigger than that of the hospital under consideration, is smaller than or equal to 0.05/number of hospitals to be compared (the so-called Bonferroni correction for multiple comparisons[23]); 2) "moderate" if that probability is smaller than or equal to 0.05 but greater than 0.05/number of hospitals to be compared; 3) "weak" otherwise.

### Study goals

The results of the analysis had to serve three purposes: to deliver (1) a feedback to the hospitals so they can improve care processes, (2) a feedback to the Belgian College of Physicians that will enable them to support hospitals identified with higher or lower quality, and (3) a feedback to health authorities as a useful tool for policy making.

The feedback to the hospitals consists mainly of a graphical display of the "departure" of all of the hospitals from the national rate/trend, of an anonymous and tabular representation of these departures as well as of an indication of the level of statistical evidence. An aid in the interpretation, combining the information of both the period and trend analyses, is provided alongside. Its decision tree is given in the Annex of the Supplementary materials (see Additional file 1).

In the feedback to the Belgian College of Physicians we present an average and two outlying categories of hospitals. A first, outlying category, the 'high CSR' group, consists of those hospitals with a departure of > +35 and statistically significant (Bonferroni-corrected). A second, outlying category, the 'low CSR' group, consists of those hospitals with a departure of < -25 and statistically significant (Bonferroni-corrected). The other hospitals are grouped into the 'average CSR' group. The decision tree is identical to that for the hospitals, except that hospitals recommended for an external audit are now divided in "high CSR" and "low CSR" groups and that the other hospitals are regrouped in an "average CSR" group.

### Neonatal endpoints

As the optimal CSR is unknown, we compared the three CSR groups with respect to a number of selected neonatal endpoints like respiratory distress syndrome (RDS), meconium aspiration syndrome (MAS) and transient tachypnea of newborn (TTN), the main causes of neonatal morbidity [24,25]. We selected 1-minute and 5-minute Apgar scores [26]; RDS, MAS, TTN (ICD-9-CM codes 769, 770.1 and 770.6); need of respiration sustaining treatments; and admission into a specialized neonatal service as neonatal endpoints. Notice that in our view 1-minute Apgar scores, essentially indicating fetal distress, are rather used as a process, reflecting the degree of accordance of the obstetrical care to fetal status, than as an outcome indicator. The 5-minute Apgar scores would allow the identification of cases vaginally delivered that might have benefited of a CS.

In order to reduce inter-observer variability and according to the literature we regrouped the Apgar scores into three categories: "Apgar 0–3," "Apgar 4–6," and "Apgar > 6" [27,28]. The completeness of the data regarding Apgar scores may be more secure than that of the other neonatal endpoints since they are recorded by means of explicitly to-be-filled-out items whereas the others are open-question-like observations a hospital may or may not register.

To determine the possible influence of major congenital anomalies on the perinatal endpoints (see Additional file 2), we planned to twice carry out our analyses: once including and once excluding the cases of congenital anomalies [29].

### Statistical methods

For our analyses we used so-called fixed effects models, because we focused on the whole of the Belgian hospitals and aimed at the identification of outlying hospitals, i.e. characterized by an important and statistically significant, Bonferroni-corrected [23] departure from the national CSR or CSR evolution over time. Given we cover the short time span of eight semesters, we only fitted models with a linear time trend, which for convenience we called "trend."

Hierarchical models, usually taking the form of so-called random-effects models, would have been an alternative. However, these models are not conceived to identify outliers. Further, the theory dealing with outliers still has to be developed for linear mixed models and it is impossible to identify outliers in non-linear mixed models [30,31]. Finally, in the random-effects models the hospitals in the set of data are considered a random sample from the larger population of all hospitals, contrary to the facts in our study.

Logistic regression [32] was performed to compare each individual hospital with all Belgian hospitals and to determine both a practically relevant and statistical significant departure from the national rate/trend. By incorporating an interaction term in the logistic regression between a linear time trend, expressed in semesters, and individual hospitals, those hospitals with an abnormal evolution in time were identified. Precisely, we compared the slope of each hospital's time trend with that of the national trend using linear contrasts.

In case of common outcomes (> 10%), or if the odds ratio is greater than 2.5 or less than 0.5, the estimation of the relative risk by the odds ratios, provided by the logistic regression, may become heavily biased. To reduce this bias we used the approximation of the RR by Zhang[33], which has been used to compute the afore-mentioned departure. The relation between RR_Z _and the odds ratio is given by RR_Z _= OR/((1-P_0_)+(P_0 _*OR), where P_0 _indicates the incidence of the outcome of interest in the non-exposed group [33].

In the main analysis, adjustment was made for age of the mother and per-semester evolution of the CSRs. In a secondary analysis, type of hospital (private versus public, teaching versus non-teaching), gestational age, admission day and residence of the mother were considered determinants, susceptible both to influence a hospitals' CSR and to be modified by quality-directed initiatives.

To account for correlation within the data, rescaling techniques were used [34]. To study a possible national upward trend we used so-called Generalized Estimating Equations (GEE), a refinement of logistic regression that corrects for correlation within the data [31].

The neonatal endpoints were analyzed by multivariable logistic regression as well. As the Apgar categories constitute a multinomial endpoint, we intended to fit a proportional odds logistic model, provided the assumption of a constant odds ratio was met [35]. Otherwise, a generalized logit analysis was to be conducted [35].

Independence of two variables forming a contingency table and proportions were analyzed by means of chi-square tests. Cochran-Armitage trend tests were used where appropriate.

These statistical analyses were performed using SAS version 8.1, SAS Institute Inc., SAS Campus Drive, Cary, North Carolina 27513, US.

The study being (1) of a retrospective, non-interventional type and (2) anonymous with respect to both hospitals and patients, no approval by an ethics committee is required in Belgium.

## Results

### Completeness and accuracy of the data

According to residence of the mother, the MCD data showed very similar to those of the NIS, indicating their high degree of completeness regarding the number of live births (Table 1, section 1). As to the neonatal characteristics we observed an acceptable agreement between MCD and SPE regarding multiple gestation, gestational age, cesarean delivery, presentation, weight at birth, gender and Apgar scores (Table 1, section 2). However, regarding hypertension, diabetes, labor induction, epidural anesthesia, and history of a previous cesarean delivery, we found important disagreements. For the years 2001 and 2003 (see Additional file 3) almost identical figures were observed. The comparison of the MCD data with those of the ONE (data not shown) showed a good agreement with respect to birth weight and Apgar scores as well.

**Table 1:** Completeness and accuracy of the data (in %): comparison between MCD versus NIS, and between MCD versus SPE

1) Comparison between MCD* and NIS°: number and distribution of live born infants, according to residence				
	**2002**		**2004**	

**Residence**	**MCD**	**NIS**	**MCD**	**NIS**

Belgium	(N = 111,609)	(N = 111,225)	(N = 116,142)	(N = 115,618)
Flanders	53.1	53.7	53.0	53.9
Wallonia	33.2	33.8	32.4	32.9
Brussels	13.1	12.5	13.8	13.1
Abroad	0.6	-	0.8	-

**2) Comparison between MCD and SPE^+^: perinatal characteristics**				

	**2002**		**2004**	
**Deliveries**	**MCD**	**NIS**	**MCD**	**NIS**
	(N = 58,194)	(N = 58,841)	(N = 59,126)	(N = 61,647)
Multiple gestation				
twins	2.3	2.0	2.0	1.6
triplets	0.1	0.0	0.1	0.0
Hypertension	5.5	4.9	5.8	4.8
Diabetes	1.6	1.2	2.1	1.4
Gestational age				
20–31 weeks	1.0	1.0	1.1	1.1
32–36 weeks	6.1	6.3	6.4	6.5
>= 37 weeks	92.9	92.7	92.8	92.5
Labor induction	19.4	30.1	17.7	27.6
Epidural anesthesia	48.3	63.2	51.3	61.6
Cesarean delivery	18.1	17.7	18.5	18.3
Previous cesarean	4.6	7.6	5.7	8.2
**Births**	**MCD**	**NIS**	**MCD**	**NIS**
	(N = 58,529)	(N = 60,048)	(N = 59,110)	(N = 62,657)
Presentation				
breech	4.5	5.4	4.5	5.2
transverse	0.5	0.6	0.4	0.5
Type of birth				
spontaneous	72.5	69.9	71.3	70.2
vacuum	8.5	10.3	9.1	9.7
forceps	0.6	1.1	0.5	0.9
cesarean	17.9	18.3	18.2	18.9
vaginal breech	0.5	0.4	0.4	0.3
Weight at birth				
<1500 g	0.8	1.2	0.8	1.1
1500–2499 g	5.6	6.2	5.4	5.8
>= 2500 g	93.3	92.7	93.7	93.1
Gender				
male	51.4	51.4	51.4	51.4
female	48.6	48.6	48.6	48.6
Congenital anomalies	3.0	1.8	3.1	1.6
Admission into specialized unit	17.9	17.9	17.9	17.8

Sources

*MCD: Minimal Clinical Data

°NIS: National Institute of Statistics

^+^SPE: Studiecentrum Perinatale Epidemiologie

### Low risk pregnancies

Comparing the study population (381,988 deliveries from which 12.98% CS) with the comparison population (73,944 deliveries of live born singletons (in vertex position) from which 49.68% CS), we found in the latter population a relative risk of being delivered by CS of 3.83, 95% CI (3.79; 3.87). Applying to our source population the basic triad of "mothers with singleton, full-term (37 weeks and more) births involving a vertex presentation", recently used to describe maternal risk profiles[21], we would have had 395,021 low risk deliveries, giving rise to 52,611 CS and to a relative risk of 1.12, 95% CI (1.10; 1.13).

### Characteristics of the study population and mode of delivery. Belgium 2001–2004

In the univariate analyses (see Additional file 4) several determinants of the CS rate were identified with relevance for the final analysis (Table 2). Increasing maternal age was associated with increasing CSRs (Cochran-Armitage: p < 0.01). We further found that the CSRs were not homogeneously distributed across birth weight and gestational age groups (Chi-square: p < 0.01), and in particular that a GA of >36 and <39 weeks was associated with the highest gestational age-specific cesarean risk as was the birth weight category of 4000–4499 g. The CSR tended to be significantly higher for boys as compared to girls (OR: 1.14; 95% CI [1.12; 1.16]) as well. We also established an increasing CSR trend and an inverse correlation between the CS rate and the 1' and 5' Apgar scores (Cochran-Armitage trend: p < 0.01). The respiratory syndromes were all significantly associated with the CSR: RDS, wet lung, and MAS had odds ratios of respectively: 3.07, 95% CI (2.76; 3.44); 3.24, 95% CI (2.93; 3.59) and 1.60, 95% CI (1.45; 1.77). Similarly, neonatal interventions such as respiratory support and admission to a specialized neonatal service were significantly associated with cesarean rates (OR2.66, 95% CI (2.47; 2.87) and 1.27, 95% CI (1.24; 1.30)). Finally, congenital anomalies proved another determinant of the CSR (OR 1.35, 95% CI (1.45; 1.77).

**Table 2:** Study population: characteristics and mode of delivery. Belgium 2001–2004.

	**CS**	**%**	**Total**		**CS**	**%**	**Total**
**1) Maternal**				**2) Neonatal**			
**Age classes**				**Gender**			
< 20 years	765	9.40	8,140	Boys	26,862	13.69	196,191
20–24 years	5,910	10.37	57,010	Girls	22,713	12.23	185,755
25–29 years	15,987	11.92	134,071	Undetermined	3		23
30–34 years	17,342	13.75	126,098	**Birth weight**			
35–39 years	7,746	16.27	47,613	2500–2999 g	9,444	13.78	68,511
40 years+	1,828	20.18	9,057	3000–3499 g	20,431	12.18	167,699
**Admission Day**				3500–3999 g	15,119	12.89	117,250
Monday	9,560	14.84	64,422	4000–4499 g	4,584	16.07	28,529
Tuesday	8,827	13.80	63,948	**Gestational age group**			
Wednesday	8,780	14.22	61,762	37–38 weeks	18,531	18.77	98,712
Thursday	8,961	14.29	62,729	39–40 weeks	25,628	10.56	242,645
Friday	5,212	10.73	48,555	41–42 weeks	5,419	13.34	40,632
Saturday	2,441	7.21	33,836	**1 min Apgar**			
Sunday	5,797	12.40	46,737	Missing	10	16.13	62
**Semester**				< 4	1,141	21.61	5,281
2001-1	5,672	11.99	47,287	4 – 6	2,857	14.65	19,507
2001-2	5,975	12.43	48,058	> 6	45,570	12.76	357,139
2002-1	5,757	12.51	46,023	**5 min Apgar**			
2002-2	6,254	13.14	47,605	Missing	10	15.38	65
2003-1	6,161	13.27	46,421	< 4	163	21.73	750
2003-2	6,550	13.55	48,356	4 – 6	703	18.03	3,898
2004-1	6,319	13.24	47,734	> 6	48,702	12.91	377,276
2004-2	6,890	13.64	50,505	**Respiratory Syndromes**			
				RDS	480	31.27	1,535
				Wet lung	552	32.43	1,702
				Meconium Aspiration	510	19.23	2,652
				**Intubation/Ventilation**	959	27.88	3,440
				**Congenital Anomaly**	559	16.57	3,374
				Admission N*/NICU	9,850	15.31	64,326

CS: number of cesarean sections carried out; %: proportion of the cesarean deliveries expressed as a percentage; Total: total number of deliveries with the characteristic under study.

With regard to health services characteristics, interestingly we documented a considerably strong and statistically significant (Chi-square: p < 0.0001) association between day of admission and both the number and proportion of cesarean deliveries, varying respectively between 64,422 and 14.84% on Monday; and, 33,836 and 7.21% on Saturday.

### Inter-hospital differences in CSR

**Figure 1:**
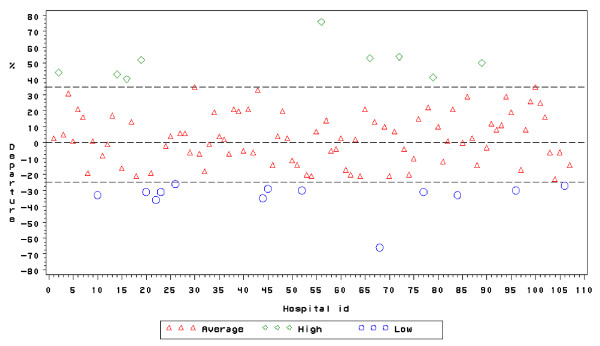
Inter-hospital differences in CSR, period. Belgium, 2001–4.

**Figure 2:**
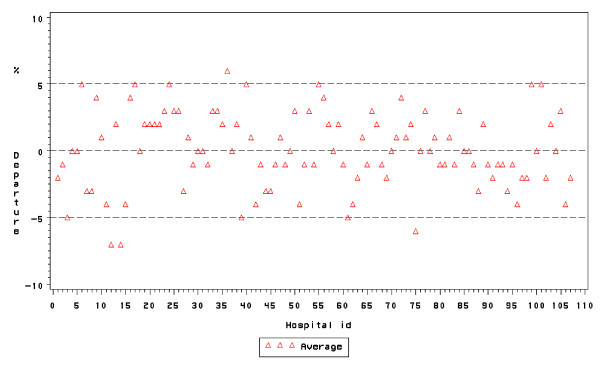
Inter-hospital differences in CSR, trend. Belgium, 2001–4.

We observed considerable and statistically significant inter-hospital differences in CSRs both in the overall analysis and in the longitudinal analysis (Figures 1 and 2). Regarding the period analysis, departures ranged from -65 up to +75, corresponding to nine "high CSR" and thirteen "low CSR" outlying hospitals with CSRs of respectively 19.3% and 8.8% vs 12.9% in the "average CSR" group. Regarding the trend, hospital-specific departures from the national CSR trend ranged from -6 to +6 and, a difference that did not achieve statistical significance when accounting for multiple comparisons. For instance, the departure, of hospital ID 36 amounted to + 6 (Bonferroni-corrected 95% CI: -2 to +14), corresponding to a p-value of 0.01245 which does not reach the Bonferroni-corrected significance level of 0.05/107 = 0.00047.

Apart from the figures, the feedback to the hospitals included a table, mentioning the departures of period and trend, as well as their Bonferroni-corrected and 95% CI, the corresponding p-values, and an aid to the interpretation. Regarding the first ten hospitals we provide an example of this feedback in Table 3.

**Table 3:** Departure of a hospital's CSR (expressed as a percentage) from the national CSR or trend, Belgium, 2001–4.

**ID**^1^	**Period**								**Trend**								**IP**^10^
	LB_B^2^	LB_95^3^	**D(%)**^4^	UB_95^5^	UB_B^6^	CI_95^7^	CI_B^8^	P^9^	LB_B^2^	LB_95^3^	**D(%)**^4^	UB_95^5^	UB_B^6^	CI_95^7^	CI_B^8^	P^9^	

**1**	-18	-10	3	16	28	NS	NS	0.68283	-11	-7	**-2**	4	8	NS	NS	0.51065	GM
**2**	24	33	44	56	66	+	+	<0.00001	-8	-5	**-1**	3	6	NS	NS	0.57928	**EA**
**3**	-20	-10	5	23	38	NS	NS	0.49978	-16	-11	**-5**	2	8	NS	NS	0.18024	**GM**
**4**	9	18	31	44	56	+	+	<0.00001	-8	-4	**0**	5	9	NS	NS	0.90712	**GM**
**5**	-20	-11	1	15	27	NS	NS	0.88383	-10	-6	**0**	5	10	NS	NS	0.93539	**GM**
**6**	9	14	21	29	35	+	+	<0.00001	0	2	**5**	8	10	+	NS	0.00045	**GM**
**7**	0	7	16	26	35	+	NS	0.00061	-9	-7	**-3**	1	4	NS	NS	0.11081	**GM**
**8**	-32	-26	-19	-11	-5	-	-	<0.00001	-10	-7	**-3**	1	4	NS	NS	0.10727	**GM**
**9**	-12	-7	1	10	17	NS	NS	0.75745	-2	1	**4**	8	11	+	NS	0.01709	**GM**
**10**	-45	-40	-33	-26	-19	-	-	<0.00001	-7	-3	**1**	6	9	NS	NS	0.67442	**EA**

^1 ^ID: Anonymous hospital identifier;

^2 ^LB_B: Lower bound of the Bonferroni confidence interval;

^3 ^LB_95: Lower bound of the 95% confidence interval;

^4 ^D: departure of a hospital's CSR compared with the national CSR/national trend;

^5 ^UB_95: Upper bound of the 95% confidence interval;

^6 ^UB_B: Upper bound of the Bonferroni confidence interval;

^7 ^CI_95: +: a 95% CI suggests a significantly higher hospital's rate/trend compared with the national rate/trend,

-: a 95% CI suggests a significantly lower hospital's rate/trend compared with the national rate/trend,

NS: a 95% CI does not suggest a significant departure of the hospital's rate/trend compared with the national rate/trend.

^8 ^CI_B: +: a Bonferroni-corrected CI strongly suggests a significantly higher hospital's rate/trend compared with the national rate/trend,

-: a Bonferroni-corrected CI strongly suggests a significantly lower hospital's rate/trend compared with the national rate/trend,

NS: a Bonferroni-corrected CI does not suggest a significant departure of the hospital's rate/trend compared with the national rate/trend.

^9 ^P value: Probability of observing departures such as these assuming the null hypothesis of no departure is true.

^10^IP: Interpretation: EA: External audit and general measures recommended; GM: General measures recommended.

### National CSR and its determinants

To assess the evolution of the CSR, we fitted a multivariable GEE model, accounting for maternal age, wherein evolution over time of the CSR was fitted as a linear trend. Starting from the first semester of 2001 we found a national increasing trend of CS of about 2% by semester (Table 4, section a). To assess the determinants as available in our data set, a second multivariable GEE was fitted, thereby accounting for time and maternal age as covariates, and determinants listed in table 4. Compared to cesarean risk associated with hospital admission on Sunday, we observed increased CSRs in case of an admission from Monday to Thursday, while the cesarean risk again decreased with admissions on Friday and Saturday. Deliveries at 37–38 weeks and at 41–42 weeks were associated with a significantly increased CSR as compared to 39–40 weeks as well, whereas we were not able to detect a significant association between type of ownership or teaching status of the hospitals and mode of delivery.

**Table 4:** CSR evolution and determinants. Belgium 2001–4.

	**OR**	**P**
**a) National trend adjusted for age group**		
**Semestrial trend **(basis 1st semester 2001)	1.02 (1.02; 1.02)	<0.01
*Age group*		
20–24 years vs < 20 years	1.11 (1.11; 1.11)	<0.01
25–29 years vs < 20 years	1.30 (1.30; 1.30)	<0.01
30–34 years vs < 20 years	1.53 (1.53; 1.53)	<0.01
35–39 years vs < 20 years	1.86 (1.86; 1.86)	<0.01
40 years+ vs < 20 years	2.41 (2.41; 2.41)	<0.01
		
**b) National trend of CSR and determinants**		
**Semestrial trend **(basis 1st semester 2001)	1.02 (1.01; 1.02)	<0.01
*Age group*		
20–24 years vs < 20 years	1.12 (1.09; 1.22)	0.02
25–29 years vs < 20 years	1.31 (1.20; 1.43)	<0.01
30–34 years vs < 20 years	1.55 (1.42; 1.69)	<0.01
35–39 years vs < 20 years	1.89 (1.73; 2.07)	<0.01
40 years+ vs < 20 years	2.45 (2.21; 2.71)	<0.01
**Gestational age**		
37–38 weeks vs 39–40 weeks	1.93 (1.87; 2.00)	<0.01
41–42 weeks vs 39–40 weeks	1.31 (1.26; 1.36)	<0.01
**Residence**		
Antwerp vs National	1.00 (0.94; 1.05)	0.73
Brussels vs National	0.89 (0.84; 0.94)	<0.01
Brabant (F) vs National	0.97 (0.91; 1.05)	0.29
Brabant (W) vs National	0.82 (0.75; 0.89)	<0.01
West Flanders vs National	1.10 (1.02; 1.18)	0.01
East Flanders vs National	1.06 (1.00; 1.13)	<0.01
Hainaut vs National	0.94 (0.88; 1.00)	<0.01
Liège vs National	1.34 (1.25; 1.44)	<0.01
Limburg vs National	1.24 (1.14; 1.34)	<0.01
Luxembourg vs National	1.01 (0.86; 1.18)	0.92
Namur vs National	0.83 (0.77; 0.90)	<0.01
Abroad vs National	0.93 (0.79; 1.09)	0.20
**Admission day**		
Monday vs Sunday	1.22 (1.15; 1.29)	<0.01
Tuesday vs Sunday	1.13 (1.06; 1.19)	<0.01
Wednesday vs Sunday	1.17 (1.10; 1.23)	<0.01
Thursday vs Sunday	1.17 (1.11; 1.24)	<0.01
Friday vs Sunday	0.85 (0.80; 0.90)	<0.01
Saturday vs Sunday	0.55 (0.52; 0.59)	<0.01

OR: odds ratio; () 95% confidence interval;

### Neonatal outcomes

The bivariate distribution of the neonatal endpoints, according to mode of delivery and CSR group is given in Table 5. With the exception of RDS after a vaginal delivery, all the endpoints were unevenly distributed (p < 0.01) across CSR groups. 1- and 5-minute "Apgar 0–3" scores were more frequent in case of CS and in the "low CSR" group. However, note that the proportion of these scores in the "low CSR" group versus "high CSR" group is smaller in case of a vaginal delivery than after a CS. Regarding RDS and MAS, we observed in the three CSR groups almost identical incidences after a vaginal delivery. The incidences after CS, however, were significantly dissimilar. Indeed, the "average CSR" group seemed to have a smaller RDS incidence, whereas a smaller MAS incidence seemed to prevail in the "high CSR" group. TTN seemed to be less problematic in the "high CSR" group, regardless of the mode of delivery. In the "low CSR" group, the need for respiratory assistance seemed more important in both modes of delivery.

**Table 5:** Neonatal endpoints according to mode of delivery and CSR group. Belgium 2001–4.

		**CSR groups**							
		**High**	**(%)**	**Average**	**(%)**	**Low**	**(%)**	**Total**	**P**

**1 min Apgar**	**Vaginal**								
	< 4	299	1.05	3,169	1.22	672	1.52	4,140	<0.01
	4 – 6	1,512	5.31	12,857	4.95	2,281	5.16	16,650	
	>6	26,680	93.64	243,671	93.83	41,218	93.31	311,569	
	**CS**								
	< 4	96	1.41	918	2.38	127	2.98	1,141	<0.01
	4 – 6	349	5.14	2,223	5.77	285	6.68	2,857	
	>6	6,351	93.45	35,366	91.84	3,853	90.34	45,570	
**5 min Apgar**	**Vaginal**								
	< 4	32	0.11	478	0.18	77	0.17	587	<0.01
	4 – 6	180	0.63	2,533	0.98	482	1.09	3195	
	>6	28,279	99.26	256,686	98.84	43,609	98.73	328,574	
	**CS**								
	< 4	11	0.16	135	0.35	17	0.40	163	<0.01
	4 – 6	51	0.75	564	1.46	88	2.06	703	
	>6	6,734	99.09	37,808	98.18	4,160	97.54	48,702	
**RDS**	**Vaginal**								
	Absent	28,405	99.68	258,913	99.69	44,038	99.66	331,356	0.73
	Present	90	0.32	816	0.31	149	0.34	1,055	
	**CS**								
	Absent	6,718	98.84	38,174	99.12	4,206	98.57	49,098	<0.01
	Present	79	1.16	340	0.88	61	1.43	480	
**MAS**	**Vaginal**								
	Absent	28,331	99.42	257,974	99.32	43,964	99.50	330,269	<0.01
	Present	164	0.58	1,755	0.68	223	0.50	2,142	
	**CS**								
	Absent	6,744	99.22	38,116	98.97	4,208	98.62	49,068	<0.01
	Present	53	0.78	398	1.03	59	1.38	510	
**TTN**	**Vaginal**								
	Absent	28,425	99.75	258,814	99.65	44,022	99.63	331,261	<0.01
	Present	70	0.25	915	0.35	165	0.37	1,150	
	**CS**								
	Absent	6,740	99.16	38,082	98.88	4,204	98.52	49,026	<0.01
	Present	57	0.84	432	1.12	63	1.48	552	
**Intubation/Ventilation**	**Vaginal**								
	Absent	28,295	99.30	257,854	99.28	43,781	99.08	329,930	<0.01
	Present	200	0.70	1,875	0.72	406	0.92	2,481	
	**CS**								
	Absent	6,670	98.13	37,804	98.16	4,145	97.14	48,619	<0.01
	Present	127	1.87	710	1.84	122	2.86	959	
**Admission to specialized neonatal service**	**Vaginal**								
	Absent	27,818	97.62	212,951	81.99	37,166	84.11	277,935	<0.01
	Present	677	2.38	46,778	18.01	7,021	15.89	54,476	
	**CS**								
	Absent	6,459	95.03	29,950	77.76	3,319	77.78	39,728	<0.01
	Present	338	4.97	8,564	22.24	948	22.22	9,850	

(%): Column percentage

The discrepancies regarding the distribution of newborns admitted into a specialized service were striking: the incidences of transfer in the "high CSR" group were five times smaller, regardless of mode of delivery. However, one should be very cautious regarding the variable "admitted into a specialized neonatal service". Indeed, according to this variable, in the "high CSR" group 342 out of 395 newborns with a 1-minute Apgar of 0 to 3 and 1713 out of 1861 newborns with a 1 minute Apgar of 4 to 6 would not have been admitted into a specialized service, which seem hardly plausible figures.

Since the proportional odds assumption was not met, a general logit analysis was carried out to analyze the association between mode of delivery, CSR group and 1-minute Apgar and 5-minute Apgar scores in a more formal way. In the 1-minute Apgar analysis, it appeared that there were significantly fewer cases of "Apgar 0–3" scores in the "high" CSR group than in the "average" CSR group, which in turn was significantly less associated with "Apgar 0–3" scores than the "low CSR" group (Table 6). Regarding the "Apgar 4–6" scores we no longer found a significant difference between "average" CSR group and "high" CSR group, whereas the difference between "average CSR" and "low CSR" groups remained significant. Note that cesarean delivery and male gender were negatively associated with the Apgar scores. The 5-minute Apgar analysis essentially gave the same results.

**Table 6:** Association of CSR group, mode of delivery and 1-minute or 5-minute Apgar scores, adjusted for gender, age class and semester. Belgium, 2001–4.

	**Response Variable**	**OR**	**P**
**a) 1 minute Apgar**			
Male vs Female	Apgar 0–3	1.32 (1.25; 1.40)	<0.01
	Apgar 4–6	1.22 (1.19; 1.26)	<0.01
CS vs VD	Apgar 0–3	1.76 (1.59; 1.95)	<0.01
	Apgar 4–6	1.17 (1.10; 1.24)	<0.01
CSR group: High vs Low	Apgar 0–3	0.56 (0.48; 0.65)	<0.01
	Apgar 4–6	0.87 (0.80; 0.95)	<0.01
CSR group: High vs Average	Apgar 0–3	0.71 (0.48; 0.80)	<0.01
	Apgar 4–6	0.97 (0.91; 1.03)	0.33
CSR group: Low vs Average	Apgar 0–3	1.27 (1.14; 1.40)	<0.01
	Apgar 4–6	1.12 (1.04; 1.19)	<0.01
Average and CS vs High and VD	Apgar 0–3	1.22 (1.08; 1.37)	0.02
	Apgar 4–6	1.11 (1.04; 1.18)	<0.01
Average and CS vs Low and VD	Apgar 0–3	0.99 (0.90; 1.10)	0.88
	Apgar 4–6	0.94 (0.88; 1.01)	0.08
High and CS vs Low and VD	Apgar 0–3	0.82 (0.70; 0.95)	0.02
	Apgar 4–6	0.85 (0.78; 0.93)	<0.01
			
**b) 5 minute Apgar**			
Male vs Female	Apgar 0–3	1.29 (1.12; 1.49)	<0.01
	Apgar 4–6	1.31 (1.23; 1.40)	<0.01
CS vs VD	Apgar 0–3	1.84 (1.37; 2.47)	<0.01
	Apgar 4–6	1.52 (1.33; 1.74)	<0.01
CSR group: High vs Low	Apgar 0–3	0.51 (0.33; 0.79)	<0.01
	Apgar 4–6	0.45 (0.37; 0.55)	<0.01
CSR group: High vs Average	Apgar 0–3	0.53 (0.37; 0.76)	<0.01
	Apgar 4–6	0.57 (0.49; 0.68)	<0.01
CSR group: Low vs Average	Apgar 0–3	1.04 (0.79; 1.38)	0.78
	Apgar 4–6	1.27 (1.12; 1.43)	<0.01
Average and CS vs High and VD	Apgar 0–3	1.15 (0.80; 1.64)	0.45
	Apgar 4–6	1.13 (0.96; 1.33)	0.15
Average and CS vs Low and VD	Apgar 0–3	0.91 (0.69; 1.21)	0.51
	Apgar 4–6	0.89 (0.78; 1.00)	0.06
High and CS vs Low and VD	Apgar 0–3	0.79 (0.51; 1.22)	0.29
	Apgar 4–6	0.79 (0.65; 0.96)	0.02

OR: odds ratio; () 95% CI.

CS: cesarean section. VD: vaginal delivery.

We also investigated the association between mode of delivery, CSR group and respiratory syndromes, certain types of respiration sustaining treatments or the admission of the newborn into a specialized neonatal pediatric service/NICU (see Additional file 5). For all the endpoints considered, CS was significantly associated with an increased occurrence of negative outcomes. For male gender, with the exception of MAS, we observed a similar relationship.

Comparing the "high CSR" and "low CSR" with the "average CSR" group, the "low CSR" group showed an important excess of RDS cases in case of cesarean delivery.

Regarding MAS, and comparing the "low CSR" and "average CSR" groups, we found an excess of MAS cases in the "average CSR" group regardless of mode of delivery. In part, this phenomenon may be due to the adjustment by gestational age, since a significant excess of deliveries at 41 and 42 weeks was observed in the "low CSR" group (RR: 1.09; 95% CI [1.07; 1.12]). By contrast in case of a CS we observed an important excess of cases in the "low CSR" group.

Respiratory support seemed more often needed in the "low CSR" group (OR: 1.27; 95% CI [1.14; 1.43]). Neonatal admission into a specialized service seemed inversely associated with the "high CSR" (OR: 0.11; 95% CI [0.10;0. 12]) and "low CSR" (OR: 0.86; 95% CI [0.84;0. 89]) groups, especially in case of a vaginal delivery. But as afore-mentioned one should be very cautious when interpreting this endpoint.

Deliveries at 37–38 weeks and at 41–42 weeks were associated with an excess of all the considered endpoints. Comparing the CSR groups regarding the ratio of CS carried out at that gestational age over the total number of CS, we found 40.1% in the "high CSR" group versus 24.2% and 37.2% respectively in the "low CSR" and "average CSR" groups.

The relationship between gestational age and MAS was a peculiar one in the sense that it was significantly negative at a gestational age of 37–38 weeks and significantly positive at a gestational age of 41–42 weeks.

The results of our analyses excluding cases of congenital anomaly were essentially the same (see Additional file 6).

## Discussion

### Main findings

Our results suggested the existence of sizeable and nation-wide inter-hospital variations in CSRs in low-risk deliveries. They rested on a very conservative analysis and interpretational approach, consisting both in defining a zone of non-interpretation and in the use of considerable threshold values before a departure from the national rate or trend was labeled important and statistically significant. We adjusted for multiple simultaneous comparisons and for presence of correlation within the data[31,34], and provided degrees of evidence regarding the observed departure of a hospital, as well as an interpretational aid., thereby avoiding false alerts and reassurance, and allowing distinction between real differences and artefacts [36].

We observed an evolution over time of the CSR, which was best summarized by a national upward trend of 2% by semester. We observed that obstetrical intervention drastically pervaded childbirth as is reflected by the geographical and hospital-related distributions of CSR, and the distribution of number and mode of deliveries by admission day [37]. Structural issues (nurse staffing, availability of physicians and anesthesiology), not registered in the MCD, may also have intervened in the decision towards elective cesarean section [38].

### Main limitations of the study

Limitations in completeness and accuracy are intrinsic to vital-statistics and administrative data [22,39] Our labor-induction, epidural anesthesia and history of a previous cesarean data are incomplete, and we observed a possible over-registration of hypertension and diabetes as well (Table 1). The miscoding of these conditions may induce serious flaws in the inter-hospital comparison. Indeed, each of them is related with higher CSRs and may reflect differences in medical or coding practices across hospitals. Therefore, given the magnitude both of the occurrence of these conditions and of the miscoding, we omitted them in the definition of our study population, whereas we used the term screening for inter-hospital differences, which should be completed by external or internal audits. Owing to the very nature of our data, we were unable to formally distinguish between primary elective and repeat cesareans. This can be viewed as another limitation, and, while further analyses of the primary elective cesareans are of major interest as they are the first starting point to contain rising CSRs, the joint analysis is of use for the research question central in this work. Consequently, to avoid flawed inter-hospital comparisons and to improve the effectiveness of the CSR as a quality indicator, multi-faceted actions such as dissemination of the present results in the hospitals, the adoption of explicitly to-filled-out items, quality control of the data and audits are required.

Also our definition of a low-risk group, building on the definition from the AHRQ[1], which includes the basic triad of "mothers with singleton, full-term (>37 weeks) births involving a vertex presentation[21]," may have been incomplete. Kabir et al. for instance used more elaborated selection criteria, based on ICD-9CM codes[40], including diabetes, hypertensive disorders, placenta previa and certain congenital anomalies. Although the medical necessity of systematically carrying out a CS in case of diabetes without macrosomia [41] and hypertensive disorders (except some cases of eclampsia with acute fetal distress persisting beyond 10–15 minutes) [42] has not yet univocally been established, current practices are associated with higher caesarean rates. Conversely, mothers suffering from pathologies such as placenta previa and congenital anomalies may be considered at risk of rightly undergoing a CS.

Due to weak case identification from administrative data [17,36], we adopted an intermediate position to define our study population. Yet, it may be acknowledged that applying our criteria to our source population instead of the basic triads criteria would have resulted in a further 12% risk reduction.

A further shortcoming of our study is the absence of maternal endpoints. Although severe under-registration has been observed in several countries in Europe [43], the maternal mortality rate may be useful in inter-country comparisons, but the low incidence of maternal deaths, 8 cases in our study population out of 12 cases in the population of live born infants, prevent inter-hospital comparisons [43]. Maternal morbidity, the other maternal endpoint has not yet been defined clearly, though probably a very useful indicator of obstetric care [43]. Conditions with permanent disability of the mother such as infertility, vaginal fistulae are exceptional in Europe [43]. "Near misses" or "life-threatening events" and risks of pregnancy and childbirth-related injuries leading to urinary and fecal incontinence are considered as possible indicators for maternal morbidity, which are to be made operational in useful indicators [43]. However, these indicators, when based on administrative data, are akin to patient safety indicators type and may share its limitations, i.e., although they are appropriate for internal quality improvement efforts, the validity of their use for comparative inter-hospital purposes is still to be established [44].

Finally, part of the limitations of administrative data may be due to the basic tension which exists between using the same data for reimbursement and for measuring quality. "When the use is reimbursement, there is a tendency to perform coding quickly and to maximize the coding of complications and co-morbidities. When the use is to assess quality, however, it is important for coders to have a complete record and to restrict diagnosis coding to conditions that affect patient care [45]." For instance, hypertension and diabetes may intervene in the algorithm used to determine the case mix of an admission and thus be rewarding in financial terms, whereas this may not be the case for labor induction, epidural anesthesia and history of a previous cesarean.

### Main strengths

The comparison of the data regarding selected variables in our study population and the data from other sources, presented in Table 1, showed a good match in terms of completeness and accuracy. Research has shown that a CS is almost always correctly classified [39]. An analysis based on a limited number of variables with known reliability may achieve an inter-hospital comparison, reasonably similar to a comparison based on medical record data [46].

### Neonatal endpoints

We did not include neonatal mortality as an endpoint because it is dependent on neonatal care, which is outside the scope of this study, and further because neonatal mortality is both a rare phenomenon and, due to the mode of registration in Belgium, cannot be linked to the type of delivery in case of transfer to another hospital.

Information on Apgar scores, in contrast, is rarely missing: 0.39% in an important Swedish study [47] and even less in ours. For the time being, we lack undisputed and countrywide accessible alternatives [47,48]. In addition, it is doubtful whether a similar degree of completeness is present regarding the other neonatal endpoints. Since we excluded multiple births, cases of prematurity, IUGR and malpresentation (including breech) [29,47,49] from our study, part of the risk factors for respiratory distress have been avoided. Unfortunately we were not able to take into account other sources of less good Apgar scores [50].

The association of high CSRs with less 1-minute "Apgar 0–3" scores seems in accordance with CS performed for fetal distress and to plead in favor of this group. However, SES-related confounding may have played a role in this relationship [50]. In this group we observed a relative excess of these scores after a vaginal delivery, whereas one would rather have expected an excess of such scores after a CS. This finding may indicate a problem of over-use, especially in this group. More generally indeed, 45,104 newborns, not suffering from congenital anomalies and having a 1-minute Apgar > 6, were delivered by CS. Since evidence from Flanders, the Northern part of Belgium, shows that about 8% of the women delivering during the study period had a history of a previous CS[51], one may conclude that at least an important part of them has been delivered by a CS not carried out for strictly medical reasons.

On the other hand, 574 newborns without congenital anomalies and with 5-minute "Apgar 0–3" scores were vaginally delivered. Similarly 3,114 of such newborns with 5-minute "Apgar 4–6" scores were delivered vaginally. Both groups might have benefited from a CS, indicating a possible under-use of this procedure. Indeed, 5-minute Apgar scores are still considered a valid predictor of neonatal mortality [28].

As in the diagnostic area and in peer review [52], arguably Apgar scores are subject to inter-observer variability [49]. But we may have removed some of it through the categorization of the Apgar scores in agreed on classes.

The results regarding other neonatal endpoints were not univocal and we were anxious about the completeness of our data, given the open-ended question type of registering. Our aforementioned finding in the "high CSR" group of an important proportion of newborns with 5-minute Apgar scores < 7 and not admitted in a specialized neonatal service illustrates this incompleteness. Literature data regarding MAS, RDS and TTN showing both similarities and important dissimilarities regarding incidence of the pathologies and their association with Apgar scores or mode of delivery are further arguments in favor of this hypothesis [25,53].

Apart from these concerns, in most of these endpoints CS seemed to be associated with less desirable neonatal outcomes and "low CSR" hospitals seemed to perform less well, which is consistent with the findings of another study [38].

Some findings did not favor the "high CSR" group neither. Indeed, deliveries at 37–38 weeks were associated with both an excess of cesarean deliveries and of all the considered endpoints. This finding is consistent with the literature stating that elective CS should be carried out at 39–40 weeks of gestational age rather than at 37–38 weeks, often the case in Belgium. Indeed, at the latter gestational age, before the onset of spontaneous labor, respiratory problems more commonly occur [9]. Comparing the CSR groups regarding the ratio of cesarean deliveries carried out at that gestational age over the total number of CS, we found 40.1% in the "high CSR" group versus 24.2% and 37.2% respectively in the "low CSR" and "average CSR" groups. Of course, in the multiple analyses the fact of having undergone a CS comes on top of the often adverse effects from the other endpoints and determinants under study. These considerations suggest that the "average CSR" group might be the more adequate benchmark.

## Conclusion

Despite our efforts to reduce the limitations typical of administrative data, our results are arguably a useful "screening," which may trigger initiatives for quality-of-care improvement in the hospitals, rather than providing definitive statements at this stage [54]. Indeed, the inter-hospital differences in CSR, the national CS upward trend, the indications of over-use and under-use, the geographically different obstetric patterns and the admission day-related concentration of deliveries, whether or not by CS, are such that an explanation is overdue.

## Disclaimer

The authors of this article are responsible for its contents. No statement in this article should be construed as an official position of the Federal Service of Health, Food Chain Safety and Environment, Directorate-General for the Organization of Health Care Establishments.

## Competing interests

The author(s) declare that they have no competing interests.

## Authors' contributions

WA conceived of the study, drafted the manuscript, and participated in the design of the study and in the statistical analysis. FW was responsible for the data management and participated in the statistical analysis. GM supervised the statistical analysis and participated in the design of the study. PVR participated in the design of the study (neonatal endpoints). HV participated in the design of the study (obstetrical endpoints). JMF participated in the design of the study (obstetrical endpoints). All authors made important contributions to the interpretation of data and the redaction of the final manuscript, and approved it.

## Pre-publication history

The pre-publication history for this paper can be accessed here:



## Supplementary Material

Additional file 1 annex. Aid to the interpretation: algorithm to classify the hospitals according to their departure from the national mean and trend. Click here for file

Additional file 2 Major congenital anomalies. ICD-9-CM codes regarding major congenital anomalies potentially related to birth asphyxia. Click here for file

Additional file 3 Table 1 completeness and accuracy extended. An exhaustive version of table 1 covering now the whole period of 2001–4 instead of the years 2002 and 2004. Click here for file

Additional file 4 Table 2 characteristics supplementary. Supplementary data are provided regarding month of admission, residence of the mother, and type of hospital (MICU, Teaching, Ownership). Click here for file

Additional file 5 Respiratory syndromes and mode of delivery (ano incl). Occurrence of respiratory syndromes, respiratory support, transfer into a specialized service associated with mode of delivery and CSR group, adjusted for gender, maternal age, gestational age and semester of delivery. Cases of congenital anomaly included. Click here for file

Additional file 6 Respiratory syndromes and mode of delivery wo anomali. Occurrence of respiratory syndromes, respiratory support, transfer into a specialized service associated with mode of delivery and CSR group, adjusted for gender, maternal age, gestational age and semester of delivery. Cases of congenital anomaly excluded. Click here for file
