# Supplementary material for: Screening for inter-hospital differences in cesarean section rates in low-risk deliveries using administrative data: An initiative to improve the quality of care
Source: BMC Health Serv Res. 2008 Jan 4;8:3. doi: 10.1186/1472-6963-8-3 (PMC2266728; doi:10.1186/1472-6963-8-3)
Supplement: Additional file 1 — annex. Aid to the interpretation: algorithm to classify the hospitals according to their departure from the national mean and trend. [file 1472-6963-8-3-S1.doc]

Annex: Aid to the interpretation

Step1: Period analysis

According to the available period information, a period score is attributed.

| Departure | Bonferroni upper bound | 95% upper bound | 95% lower bound | Bonferroni lower bound | Period score |
| --- | --- | --- | --- | --- | --- |
| > + 35 |  |  |  | ≥ 0 | 1 |
| > + 35 |  |  | ≥ 0 | < 0 | 2 |
| < - 25 | > 0 | ≤ 0 |  |  | 3 |
| < - 25 | ≤ 0 |  |  |  | 4 |
| < + 36 and > -26 |  |  |  |  | 5 |
| > + 35 |  |  | < 0 |  | 6 |
| < - 25 |  | > 0 |  |  | 7 |

Step 2: Trend analysis

According to the available trend information, a trend score is attributed.

| Departure | Bonferroni upper bound | 95% upper bound | 95% lower bound | Bonferroni lower bound | Trend score |
| --- | --- | --- | --- | --- | --- |
| > + 5 |  |  |  | ≥ 0 | 1 |
| > + 5 |  |  | ≥ 0 | < 0 | 2 |
| < - 5 | > 0 | ≤ 0 |  |  | 3 |
| < - 5 | ≤ 0 |  |  |  | 4 |
| < 6 and > -6 |  |  |  |  | 5 |

Step 3: Combination of period and trend analysis

| Period score | Trend score | CSR group | Feedback |
| --- | --- | --- | --- |
| 1 | 1 | High | EA |
| 1 | 2 | High | EA |
| 1 | 3 | High | EA |
| 1 | 4 | Average | IA |
| 1 | 5 | High | EA |
| 2 | 1 | Average | IA |
| 2 | 2 | Average | IA |
| 2 | 3 | Average | Ia |
| 2 | 4 | Average | Ia |
| 2 | 5 | Average | IA |
| 3 | 1 | Average | Ia |
| 3 | 2 | Average | Ia |
| 3 | 3 | Average | IA |
| 3 | 4 | Average | IA |
| 3 | 5 | Average | IA |
| 4 | 1 | Average | IA |
| 4 | 2 | Low | EA |
| 4 | 3 | Low | EA |
| 4 | 4 | Low | EA |
| 4 | 5 | Low | EA |
| 5 | 1 | Average | IA |
| 5 | 2 | Average | Ia |
| 5 | 3 | Average | Ia |
| 5 | 4 | Average | IA |
| 5 | 5 | Average | GM |
| 6 | Any | Average | Ia |
| 7 | Any | Average | Ia |

EA: external audit recommended; IA: internal audit recommended; ia: internal audit to be considered; GM: take general measures.
