# Supplementary material for: Screening for inter-hospital differences in cesarean section rates in low-risk deliveries using administrative data: An initiative to improve the quality of care
Source: BMC Health Serv Res. 2008 Jan 4;8:3. doi: 10.1186/1472-6963-8-3 (PMC2266728; doi:10.1186/1472-6963-8-3)
Supplement: Additional file 2 — Major congenital anomalies. ICD-9-CM codes regarding major congenital anomalies potentially related to birth asphyxia. [file 1472-6963-8-3-S2.doc]

Major congenital anomalies potentially related to birth asphyxia

| Congenital anomaly | ICD−9−CM |
| --- | --- |
| Neural tube defects | 740.0−741.9 |
| Brain anomalies | 742.0, 742.2−742.4, 745.9 |
| Microcephaly | 742.1 |
| Major congenal heart disease | 745.0−745.3, 745.6, 746.0−746.9, 747.1, 747.3, 747.41 |
| Cerebrovascular anomaly | 747.81 |
| Respiratory system anomaly | 748.3−748.6 |
| Gastrointestinal anomaly | 750.3, 751.0−751.5,751.8, 751.9 |
| Renal/bladder anomaly | 753.0−753.9 |
| Cranosynostosis | 756.0 |
| Abdominal wall defect | 756.7 |
| Down syndrome | 758.0 |
| Other congenital anomalies | 758.1−758.9 |
| Multiple anmalies unspecified | 759.7 |
