# Supplementary material for: Screening for inter-hospital differences in cesarean section rates in low-risk deliveries using administrative data: An initiative to improve the quality of care
Source: BMC Health Serv Res. 2008 Jan 4;8:3. doi: 10.1186/1472-6963-8-3 (PMC2266728; doi:10.1186/1472-6963-8-3)
Supplement: Additional file 3 — Table 1 completeness and accuracy extended. An exhaustive version of table 1 covering now the whole period of 2001–4 instead of the years 2002 and 2004. [file 1472-6963-8-3-S3.doc]

Table 1: Completeness and accuracy of the data (in %). Belgium 2001-4

| **1) Comparison between MCD and NIS° of the number and distribution of liveborn infants, according to residence** | | | | | | | | | |
| --- | --- | --- | --- | --- | --- | --- | --- | --- | --- |
|  | **2001** | | **2002** | | **2003** | | **2004** | |  |
| **Residence** | **MCD** | **NIS** | **MCD** | **NIS** | **MCD** | **NIS** | **MCD** | **NIS** |  |
| Belgium | (N=113,600) | (N=114,172) | (N=111,609) | (N=111,225) | (N=112,704) | (N=112,149) | (N=116,142) | (N=115,618) |  |
| Flanders | 52.6 | 53.1 | 53.1 | 53.7 | 52.6 | 53.5 | 53.0 | 53.9 |  |
| Wallonia | 33.7 | 34.2 | 33.2 | 33.8 | 33.0 | 33.5 | 32.4 | 32.9 |  |
| Brussels | 13.2 | 12.7 | 13.1 | 12.5 | 13.7 | 13.1 | 13.8 | 13.1 |  |
| Abroad | 0.5 | - | 0.6 | - | 0.7 | - | 0.8 | - |  |
|  |  |  |  |  |  |  |  |  |  |
| **2) Comparison between MCD and SPE^+^ of perinatal characteristics** | | | | | | | | |  |
|  | **2001** | | **2002** | | **2003** | | **2004** | |  |
| **Deliveries** | **MCD** | **SPE** | **MCD** | **SPE** | **MCD** | **SPE** | **MCD** | **SPE** |  |
|  | (N=58,158) | (N=59,750) | (N=58,194) | (N=58,841) | (N=56,678) | (N=59,253) | (N=59,126) | (N=61,647) |  |
| Multiple gestation twins | 2,1 | 1,7 | 2,3 | 2,0 | 2,3 | 1,9 | 2,0 | 1,6 |  |
| triplets | 0,1 | 0,0 | 0,1 | 0,0 | 0,1 | 0,0 | 0,1 | 0,0 |  |
| Hypertension | 5,4 | 4,9 | 5,5 | 4,9 | 5,7 | 4,8 | 5,8 | 4,8 |  |
| Diabetes | 1,5 | NA | 1,6 | 1,2 | 2,1 | 1,4 | 2,1 | 1,4 |  |
| Gestational age |  |  |  |  |  |  |  |  |  |
| 20-31 weeks | 1,0 | 1,0 | 1,0 | 1,0 | 1,1 | 1,3 | 1,1 | 1,1 |  |
| 32-36 weeks | 5,9 | 6,1 | 6,1 | 6,3 | 6,4 | 6,1 | 6,4 | 6,5 |  |
| >=37 weeks | 92,9 | 92,8 | 92,9 | 92,7 | 92,6 | 92,6 | 92,8 | 92,5 |  |
| Labor induction | 19,6 | 30,7 | 19,4 | 30,1 | 18,4 | 30,0 | 17,7 | 27,6 |  |
| Epidural anesthesia | 47,8 | 62,7 | 48,3 | 63,2 | 47,1 | 64,4 | 51,3 | 61,6 |  |
| Cesarean delivery | 17,4 | 17,1 | 18,1 | 17,7 | 18,6 | 18,3 | 18,5 | 18,3 |  |
| Previous cesarean | 3,9 | 7,7 | 4,6 | 7,6 | 5,2 | 7,6 | 5,7 | 8,2 |  |
| **Births** | **MCD** | **SPE** | **MCD** | **SPE** | **MCD** | **SPE** | **MCD** | **SPE** |  |
|  | (N=58,302) | (N=60,813) | (N=58,529) | (N=60,048) | (N=56,848) | (N=60,406) | (N=59110) | (N=62,657) |  |
| Multiplets twins | 3,3 | 3,4 | 3,6 | 3,9 | 3,6 | 3,7 | 3,0 | 3,2 |  |
| >=3 | 0,1 | 0,1 | 0,1 | 0,1 | 0,1 | 0,1 | 0,1 | 0,1 |  |
| Presentation |  |  |  |  |  |  |  |  |  |
| breech | 4,5 | 5,3 | 4,5 | 5,4 | 4,5 | 5,4 | 4,5 | 5,2 |  |
| transverse | 0,5 | 0,6 | 0,5 | 0,6 | 0,4 | 0,5 | 0,4 | 0,5 |  |
| Type of birth |  |  |  |  |  |  |  |  |  |
| spontaneous | 74,3 | 70,3 | 72,5 | 69,9 | 71,8 | 70,0 | 71,3 | 70,2 |  |
| vacuum | 8,5 | 10,5 | 8,5 | 10,3 | 8,7 | 9,7 | 9,1 | 9,7 |  |
| forceps | 0,6 | 1,2 | 0,6 | 1,1 | 0,6 | 1,0 | 0,5 | 0,9 |  |
| cesarean | 16,9 | 17,6 | 17,9 | 18,3 | 18,4 | 18,9 | 18,2 | 18,9 |  |
| vaginal breech | 0,6 | 0,4 | 0,5 | 0,4 | 0,5 | 0,3 | 0,4 | 0,3 |  |
| Weight at birth |  |  |  |  |  |  |  |  |  |
| <1500 g | 0,8 | 1,1 | 0,8 | 1,2 | 0,9 | 1,2 | 0,8 | 1,1 |  |
| 1500-2499 g | 5,4 | 5,9 | 5,6 | 6,2 | 5,7 | 6,2 | 5,4 | 5,8 |  |
| >=2500 g | 93,4 | 92,9 | 93,3 | 92,7 | 93,5 | 92,7 | 93,7 | 93,1 |  |
| Gender |  |  |  |  |  |  |  |  |  |
| male | 51,4 | 51,3 | 51,4 | 51,4 | 51,2 | 51,1 | 51,4 | 51,4 |  |
| female | 48,6 | 48,6 | 48,6 | 48,6 | 48,8 | 48,9 | 48,6 | 48,6 |  |
| Congenital anomalies | 3,0 | 1,5 | 3,0 | 1,8 | 3,0 | 1,6 | 3,1 | 1,6 |  |
| Transfer into specialized unit | 18,8 | 17,1 | 17,9 | 17,9 | 18,9 | 18,2 | 17,9 | 17,8 |  |
|  |  |  |  |  |  |  |  |  |  |
| **3) Comparison of birth weight and Apgar scores distributions between MCD (2001) and published data* (2000)** | | | | | | | | |  |
|  | MCD-2001 | B3-2000 | MCD-2001 | B2-2000 |  |  |  |  |  |
| Birth weight |  |  |  |  |  |  |  |  |  |
| < 500 g | 0.02 | 0,06 | 0.02 | n/a |  |  |  |  |  |
| 500-1499 g | 0.96 | 1,18 | 0.83 | 0,9 |  |  |  |  |  |
| < 1500 g | 0.98 | 1,24 | 0.85 | 0,9 |  |  |  |  |  |
| 1500-2499 | 7.02 | 6,25 | 5.76 | 5,66 |  |  |  |  |  |
| < 2500 g | 8.00 | 7,48 | 6.61 | 6,56 |  |  |  |  |  |
| 2500-4499 g | 91.39 | 90,77 | 92.43 | 92,49 |  |  |  |  |  |
| 4500 g+ | 0.61 | 1,75 | 0.96 | 0,95 |  |  |  |  |  |
| Apgar scores |  |  |  |  |  |  |  |  |  |
| < 4 | 4.2 | 2.8 | 3.7 | 2.5 |  |  |  |  |  |
| <7 | 17.5 | 14.4 | 18.6 | 18.1 |  |  |  |  |  |

Sources: ° NIS: National Institute of Statistics;^+^ SPE: Studiecentrum Perinatale Epidemiologie;^*^ Buitendijk
