# Supplementary material for: Screening for inter-hospital differences in cesarean section rates in low-risk deliveries using administrative data: An initiative to improve the quality of care
Source: BMC Health Serv Res. 2008 Jan 4;8:3. doi: 10.1186/1472-6963-8-3 (PMC2266728; doi:10.1186/1472-6963-8-3)
Supplement: Additional file 4 — Table 2 characteristics supplementary. Supplementary data are provided regarding month of admission, residence of the mother, and type of hospital (MICU, Teaching, Ownership). [file 1472-6963-8-3-S4.doc]

Table 2: Characteristics of the study population and mode of delivery. Belgium 2001-2004.

|  | **CS°** | **%°** | **Total°** |  |  | **CS** | **%** | **Total** |
| --- | --- | --- | --- | --- | --- | --- | --- | --- |
| **1) Maternal** | |  |  |  | **2) Neonatal** | |  |  |
| **Age classes** | |  |  |  | **Gender** |  |  |  |
| < 20years | 765 | 9.40 | 8,140 |  | Boys | 26,862 | 13.69 | 196,191 |
| 20-24 years | 5,910 | 10.37 | 57,010 |  | Girls | 22,713 | 12.23 | 185,755 |
| 25-29 years | 15,987 | 11.92 | 134,071 |  | Undetermined | 3 |  | 23 |
| 30-34 years | 17,342 | 13.75 | 126,098 |  | **Birth weight** | |  |  |
| 35-39 years | 7,746 | 16.27 | 47,613 |  | 2500-2999 g | 9,444 | 13.78 | 68,511 |
| 40 years+ | 1,828 | 20.18 | 9,057 |  | 3000-3499 g | 20,431 | 12.18 | 167,699 |
| **Admission Day** | |  |  |  | 3500-3999 g | 15,119 | 12.89 | 117,250 |
| Monday | 9,560 | 14.84 | 64,422 |  | 4000-4499 g | 4,584 | 16.07 | 28,529 |
| Tuesday | 8,827 | 13.80 | 63,948 |  | **Gestational age group** | | |  |
| Wednesday | 8,780 | 14.22 | 61,762 |  | 37-38 weeks | 18,531 | 18.77 | 98,712 |
| Thursday | 8,961 | 14.29 | 62,729 |  | 39-40 weeks | 25,628 | 10.56 | 242,645 |
| Friday | 5,212 | 10.73 | 48,555 |  | 41-42 weeks | 5,419 | 13.34 | 40,632 |
| Saturday | 2,441 | 7.21 | 33,836 |  | **1 min Apgar** | |  |  |
| Sunday | 5,797 | 12.40 | 46,737 |  | Missing | 10 | 16.13 | 62 |
| **Month** |  |  |  |  | < 4 | 1,141 | 21.61 | 5,281 |
| January | 4,280 | 13.20 | 32,434 |  | 4 - 6 | 2,857 | 14.65 | 19,507 |
| February | 3,821 | 12.98 | 29,428 |  | > 6 | 45,570 | 12.76 | 357,139 |
| March | 4,032 | 12.56 | 32,112 |  | **5 min Apgar** | |  |  |
| April | 3,941 | 12.56 | 31,365 |  | Missing | 10 | 15.38 | 65 |
| May | 4,025 | 12.69 | 31,726 |  | < 4 | 163 | 21.73 | 750 |
| June | 4,109 | 12.99 | 31,626 |  | 4 – 6 | 703 | 18.03 | 3,898 |
| July | 4,357 | 12.78 | 34,093 |  | > 6 | 48,702 | 12.91 | 377,276 |
| August | 4,206 | 12.76 | 32,968 |  | **Respiratory Syndromes** | | |  |
| September | 4,319 | 13.28 | 32,519 |  | RDS | 480 | 31.27 | 1,535 |
| October | 4,114 | 12.89 | 31,923 |  | Wet lung | 552 | 32.43 | 1,702 |
| November | 4,040 | 13.40 | 30,158 |  | Meconium Aspiration | 510 | 19.23 | 2,652 |
| December | 4,334 | 13.70 | 31,637 |  | **Intubation/Ventilation** | 959 | 27.88 | 3,440 |
| **Semester** | |  |  |  | **Congenital Anomaly** | 559 | 16.57 | 3,374 |
| 2001-1 | 5,672 | 11.99 | 47,287 |  | **Admission in specialized**  **neonatal service** | 9,850 | 15.31 | 64,326 |
| 2001-2 | 5,975 | 12.43 | 48,058 |  |  |  |  |  |
| 2002-1 | 5,757 | 12.51 | 46,023 |  | **3) Hospital** | |  |  |
| 2002-2 | 6,254 | 13.14 | 47,605 |  | **Micu** | 29 | 23.82 | 1,251 |
| 2003-1 | 6,161 | 13.27 | 46,421 |  | **Ownership** | |  |  |
| 2003-2 | 6,550 | 13.55 | 48,356 |  | Public | 17,210 | 13.12 | 131,176 |
| 2004-1 | 6,319 | 13.24 | 47,734 |  | Private | 32,368 | 12.91 | 250,813 |
| 2004-2 | 6,890 | 13.64 | 50,505 |  | **Teaching** | |  |  |
| **Residence (province)** | | |  |  | Non-teaching | 43,393 | 13.03 | 332,953 |
| Antwerp | 7,411 | 12.58 | 58,924 |  | Teaching | 6,185 | 12.61 | 49,036 |
| Brabant (Flemish) | 4,492 | 12.80 | 35,098 |  | **Total** | **49,578** | **12.98** | **381,989** |
| Brabant (Walloon) | 1,476 | 11.35 | 13,009 |  |  |  |  |  |
| Brussels | 5,947 | 11.43 | 52,024 |  |  |  |  |  |
| West Flanders | 4,867 | 13.42 | 36,262 |  |  |  |  |  |
| East Flanders | 6,195 | 13.13 | 47,175 |  |  |  |  |  |
| Hainaut | 5,785 | 12.21 | 47,388 |  |  |  |  |  |
| Liège | 6,043 | 16.75 | 36,07 |  |  |  |  |  |
| Limbourg | 3,766 | 14.83 | 25,399 |  |  |  |  |  |
| Luxembourg | 1,281 | 12.69 | 10,098 |  |  |  |  |  |
| Namur | 1,920 | 11.08 | 17,326 |  |  |  |  |  |
| Abroad | 395 | 12.28 | 3,216 |  |  |  |  |  |

°CS: cesarean section; %: proportion of the cesarean deliveries expressed as a percentage;Total: total number of deliveries with the characteristic under study.
