# Supplementary material for: Screening for inter-hospital differences in cesarean section rates in low-risk deliveries using administrative data: An initiative to improve the quality of care
Source: BMC Health Serv Res. 2008 Jan 4;8:3. doi: 10.1186/1472-6963-8-3 (PMC2266728; doi:10.1186/1472-6963-8-3)
Supplement: Additional file 5 — Respiratory syndromes and mode of delivery (ano incl). Occurrence of respiratory syndromes, respiratory support, transfer into a specialized service associated with mode of delivery and CSR group, adjusted for gender, maternal age, gestational age and semester of delivery. Cases of congenital anomaly included. [file 1472-6963-8-3-S5.doc]

Table 7: Occurrence of respiratory syndromes, respiratory support, transfer into a specialized service associated with mode of delivery and CSR group, adjusted for gender, maternal age, gestational age and semester of delivery. Belgium 2001-4.

|  | **RDS** | | |  | **MAS** | | |  | TTN | | |  | **Respiratory**  **support** | | |  | **Transfer** | | |
| --- | --- | --- | --- | --- | --- | --- | --- | --- | --- | --- | --- | --- | --- | --- | --- | --- | --- | --- | --- |
|  | **OR°** | **LB°** | **UB°** |  | OR | **LB** | **UB** |  | **OR** | **LB** | **UB** |  | **OR** | **LB** | **UB** |  | **OR** | **LB** | **UB** |
| Mode of delivery |  |  |  |  |  |  |  |  |  |  |  |  |  |  |  |  |  |  |  |
| Cesarean vs Vaginal | 2.47 | 2.18 | 2.81 |  | 1.64 | 1.47 | 1.82 |  | 2.83 | 2.52 | 3.18 |  | 2.45 | 2.23 | 2.68 |  | 1.30 | 1.26 | 1.34 |
| CSR |  |  |  |  |  |  |  |  |  |  |  |  |  |  |  |  |  |  |  |
| High vs Average | 0.98 | 0.79 | 1.22 |  | 0.90 | 0.77 | 1.05 |  | 0.68 | 0.53 | 0.86 |  | 0.97 | 0.83 | 1.13 |  | 0.11 | 0.1 | 0.12 |
| Low vs Average | 1.08 | 0.90 | 1.28 |  | 0.74 | 0.64 | 0.85 |  | 1.06 | 0.90 | 1,25 |  | 1.27 | 1.14 | 1.43 |  | 0.86 | 0.84 | 0.89 |
| Gender |  |  |  |  |  |  |  |  |  |  |  |  |  |  |  |  |  |  |  |
| Male vs Female | 1.64 | 1.48 | 1.82 |  | 1.04 | 0.97 | 1.12 |  | 1.46 | 1.32 | 1.61 |  | 1.36 | 1.27 | 1.46 |  | 1.05 | 1.03 | 1.07 |
| **Maternal age** |  |  |  |  |  |  |  |  |  |  |  |  |  |  |  |  |  |  |  |
| < 20 years vs 40 years+ | 1.02 | 0.69 | 1.51 |  | 0.76 | 0.54 | 1.08 |  | 0.95 | 0.63 | 1.43 |  | 1.21 | 0.90 | 1.62 |  | 1.49 | 1.37 | 1.62 |
| 20-24 years vs 40 years+ | 0.71 | 0.53 | 0.94 |  | 0.89 | 0.70 | 1.12 |  | 0.87 | 0.66 | 1.16 |  | 0.91 | 0.72 | 1.13 |  | 1.25 | 1.17 | 1.34 |
| 25-29 years vs 40 years+ | 0.67 | 0.51 | 0.89 |  | 0.80 | 0.64 | 1.00 |  | 0.82 | 0.62 | 1.07 |  | 0.88 | 0.71 | 1.09 |  | 1.04 | 0.98 | 1.11 |
| 30-34 years vs 40 years+ | 0.71 | 0.54 | 0.93 |  | 0.79 | 0.63 | 0.99 |  | 0.77 | 0.58 | 1.01 |  | 0.84 | 0.68 | 1.04 |  | 0.92 | 0.86 | 0.98 |
| 35-39 years vs 40 years+ | 0.82 | 0.61 | 1.10 |  | 0.89 | 0.70 | 1.13 |  | 0.81 | 0.60 | 1.08 |  | 0.85 | 0.67 | 1.06 |  | 0.90 | 0.84 | 0.96 |
| **Gestational age** |  |  |  |  |  |  |  |  |  |  |  |  |  |  |  |  |  |  |  |
| 37-38 weeks vs 39-40 weeks | 2.15 | 1.93 | 2.39 |  | 0.51 | 0.46 | 0.57 |  | 2.16 | 1.96 | 2.39 |  | 1.43 | 1.33 | 1.55 |  | 1.20 | 1.17 | 1.22 |
| 41-42 weeks vs 39-40 weeks | 1.29 | 1.09 | 1.54 |  | 1.75 | 1.59 | 1.93 |  | 1.12 | 0.94 | 1.33 |  | 1.43 | 1.28 | 1.59 |  | 0.91 | 0.88 | 0.94 |
| **Semester** |  |  |  |  |  |  |  |  |  |  |  |  |  |  |  |  |  |  |  |
| 2001-1 vs 2004-2 | 1.12 | 0.93 | 1.35 |  | 0.95 | 0.83 | 1.10 |  | 0.61 | 0.51 | 0.73 |  | 1.08 | 0.94 | 1.25 |  | 1.30 | 1.26 | 1.35 |
| 2001-2 vs 2004-2 | 0.96 | 0.79 | 1.16 |  | 0.78 | 0.67 | 0.90 |  | 0.53 | 0.44 | 0.65 |  | 1.06 | 0.92 | 1.23 |  | 1.15 | 1.11 | 1.19 |
| 2002-1 vs 2004-2 | 0.81 | 0.66 | 0.99 |  | 0.83 | 0.72 | 0.97 |  | 0.65 | 0.55 | 0.79 |  | 1.28 | 1.11 | 1.47 |  | 1.03 | 0.99 | 1.07 |
| 2002-2 vs 2004-2 | 0.93 | 0.76 | 1.13 |  | 0.83 | 0.72 | 0.96 |  | 0.57 | 0.47 | 0.68 |  | 1.27 | 1.11 | 1.46 |  | 1.06 | 1.02 | 1.10 |
| 2003-1 vs 2004-2 | 1.00 | 0.82 | 1.21 |  | 0.69 | 0.60 | 0.81 |  | 0.75 | 0.63 | 0.89 |  | 1.37 | 1.20 | 1.57 |  | 1.09 | 1.05 | 1.13 |
| 2003-2 vs 2004-2 | 0.97 | 0.80 | 1.18 |  | 0.81 | 0.70 | 0.93 |  | 0.79 | 0.66 | 0.93 |  | 1.08 | 0.94 | 1.25 |  | 1.08 | 1.04 | 1.13 |
| 2004-1 vs 2004-2 | 0.90 | 0.74 | 1.09 |  | 0.99 | 0.86 | 1.14 |  | 0.87 | 0.74 | 1.02 |  | 0.95 | 0.82 | 1.10 |  | 1.03 | 0.99 | 1.07 |
| **Interaction mode of delivery and CSR group** |  |  |  |  |  |  |  |  |  |  |  |  |  |  |  |  |  |  |  |
| CS in High vs VD* in Average | 1.34 | 0.97 | 1.86 |  | 0.87 | 0.63 | 1.20 |  | 1.08 | 0.75 | 1.55 |  | 1.04 | 0.81 | 1.34 |  | 1.66 | 1.43 | 1.93 |
| CS and Low vs VD in Average | 1.53 | 1.11 | 2.12 |  | 1.74 | 1.29 | 2.36 |  | 1.26 | 0.92 | 1.72 |  | 1.23 | 0.98 | 1.55 |  | 1.18 | 1.08 | 1.29 |

°OR: odds ratio ; LB,UB: respectively lower bound and upper bound of its 95% CI. * VD: vaginal delivery. *CS: cesarean section.
